# Experimental study and analysis of lubricants dispersed with nano Cu and TiO_2 _in a four-stroke two wheeler

**DOI:** 10.1186/1556-276X-6-233

**Published:** 2011-03-17

**Authors:** Pullela K Sarma, Vadapalli Srinivas, Vedula Dharma Rao, Ayyagari Kiran Kumar

**Affiliations:** 1 GITAM University, Visakhapatnam 530045, India; 2 Andhra University, Visakhapatnam 530003, India; 3 DMSSVH College of Engineering, Machilipatnam 521002, Andhra Pradesh, India

## Abstract

The present investigation summarizes detailed experimental studies with standard lubricants of commercial quality known as Racer-4 of Hindustan Petroleum Corporation (India) dispersed with different mass concentrations of nanoparticles of Cu and TiO_2_. The test bench is fabricated with a four-stroke Hero-Honda motorbike hydraulically loaded at the rear wheel with proper instrumentation to record the fuel consumption, the load on the rear wheel, and the linear velocity. The whole range of data obtained on a stationery bike is subjected to regression analysis to arrive at various relationships between fuel consumption as a function of brake power, linear velocity, and percentage mass concentration of nanoparticles in the lubricant. The empirical relation correlates with the observed data with reasonable accuracy. Further, extension of the analysis by developing a mathematical model has revealed a definite improvement in brake thermal efficiency which ultimately affects the fuel economy by diminishing frictional power in the system with the introduction of nanoparticles into the lubricant. The performance of the engine seems to be better with nano Cu-Racer-4 combination than the one with nano TiO_2_.

## Introduction

At a very galloping speed, the human needs and demands for comforts are increasing in every corner of the world. Consequentially, the consumption of energy resources is indiscriminatingly planned without looking into the grave situation that might arise in the near future.

The increase in entropy and the environmental pollutions in every sector affect very seriously our well-being and life on this planet. The most common and the preferred mode of transportation in India is a two-wheeler, and the survey conducted by the Environment Pollution (Prevention and Control) Authority for the national capital region emphatically declared through a systematic survey that the two wheeler is the worst offender in metropolitan cities. The two-stroke engine is rated as the worst offender because of reasons: first, it emits high quantities of hydrocarbons, and second, a large quantity of the unburnt fuel is vented out. The density of two-wheeler vehicular transport increases day by day region wise in the world year after year in course of time in line with the increase in comforts and living standards of the citizens.

The prescribed emission norms for the two wheelers as per BS II standards (2005) are as follows:

CO      1.5 g/km

Hydrocarbons + NO_*x*_      1.5 g/km

However, pollution rate of CO and NO_*x *_is alarmingly much more than the prescribed norms by the Governmental agencies because of substandard manufacturing designs and improper combustion of fuel in the cylinder. In very thickly populated regions of the metropolitan cities, it definitely affects the health leading to ill health and severe respiratory problems. Besides, the fuel consumption rate is enormously high due to slow-moving two wheeler vehicular transports in busy localities. Hence, attention is bestowed to conserve the fuel for better future by employing safer alternative sources and conservation of fuel by improving overall efficiencies of the existing systems. Application of nano fluids in several engineering practices is gaining paramount importance, and in the literature, many studies related to nano tribology [1-12] can be found.

The article presents results obtained on a four-stroke two wheeler with the lubricants dispersed with nanoparticles of Cu and TiO_2 _of different mass percentage concentrations in the lubricants. The results indicate that the brake thermal efficiencies can be enhanced so that the fuel consumption rate can be improved by admixing nanoparticles into the lubricant.

### Test rig with four-stroke motorbike

The motorbike employed in the study is a four-stroke two wheeler available in the INDIAN market under the brand name HERO-HONDA. The specifications of the motor bike are as follows:

No. of strokes:      four

Diameter of the cylinder:      50 mm

Length of the stroke:      49.5 mm

Displacement volume:      97.2 cc

Air-cooled cylinder with aluminum alloy extended fins

Throttle-controlled speed regulator.

The rated brake power at the wheel is around 5.67 kW at a speed of 7,500 rpm

The recommended commercial lubricant for the motorbike is SAE 20 W 40 grade lubricant (Racer-4 of Hindustan Petroleum Corporation is suitable as engine oil).

Fuel is petrol of general quality sold in the commercial outlets situated in local areas.

### Preparation of the motorbike prior to mounting on the stand

The motorbike is a new one from the dealer, and hence initially the bike is run with lubricant Racer-4, covering a mileage of 1500 km so that the rotating and reciprocating components in the engine are well lubricated and minor manufacturing or assembly flaws can be ruled out. The bike is mounted firmly on the test platform with the front wheel firmly gripped in the special vice designed for the purpose. Besides, the frame of vehicle is vertically held in position with the rear wheel resting on two freely rotating rollers mounted on special bearings. The surface of the rollers is specially made with corrugations to avoid slipping of the rear wheel during experimentation.

**Figure 1:**
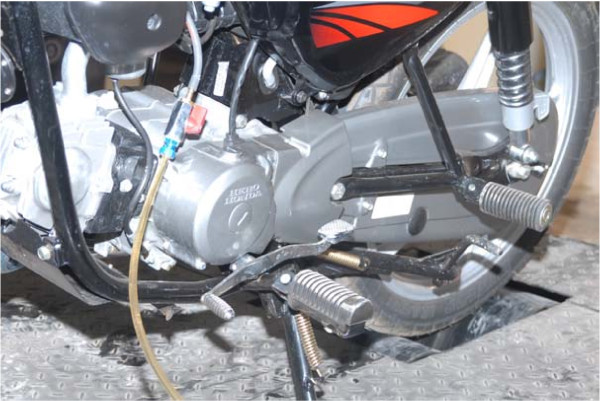
**Motorbike mounted on roller test bench**.

A hydraulic dynamometer arrangement loads the rear wheel, and its magnitude is measured with the aid of proper digital measuring device at a specific rotational speed. The fuel line to the engine is through a digital measuring device to register fuel consumption rate with good accuracy. Under running conditions, a blower is used to blow air over the finned surface of the cylinder to cool the engine-simulating actual road conditions. The cooling arrangement is made to reduce the heat buildup in the engine, preventing adverse effects on lubrication and hence the mileage. The photographic view of the rig with motorbike in position is shown in Figure 1.

The lubricant used in the bike is to lubricate the reciprocating parts like piston-cylinder and rotary parts in the gear drive. Therefore, the test procedure takes into account the sliding friction as well as gear friction and the frictional power lost in overcoming them.

The brake power can be calculated using the relation: 

#### Preparation of the nano-lubricant with the nano component

**Figure 2:**
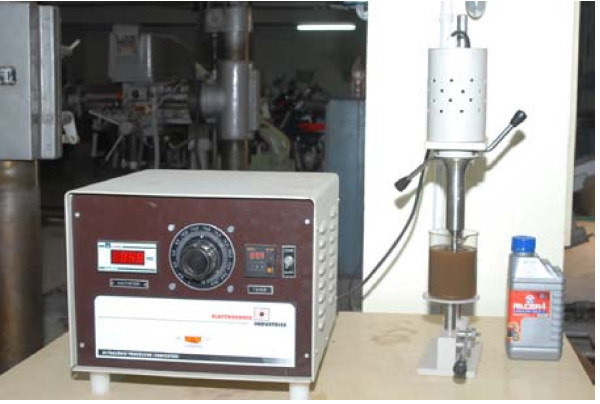
**Sonicator**.

One of the major hurdles in introducing the nano material into the racer is agglomeration, inhibiting ideal homogeneity and dispersion. An ultrasonic de-agglomerator (Sonicator) has been purchased with the following specifications to ensure homogeneous mixing and dispersion of the nanoparticles into lubricants without agglomeration (Figure 2):

Maximum power output:     600 W

Operating frequency:      20 kHz

Input:      110VAC @ 10 Amps

Programmable timer:      1 s to 1 h

The base lubricant used in the study is Racer-4 manufactured by Hindustan Petroleum Corporation Ltd., India. It is a 4-stroke bike engine oil-cum-gear oil with a grade of SAE 20W-40. Since the base lubricant is a popular commercial lubricant, it already contains some amount of dispersant, and hence in this study, no additional dispersant is added to the base lubricant. Copper (<50 nm) and titanium dioxide (<25 nm) nanoparticles are mixed in various mass fractions (0.05, 0.1, and 0.2%) into the base lubricant to prepare the required sample. The required quantity is made in batches of 400 c.c at a time with the mass of the nano material being accurately measured by electronic weighing machines with a least count of 10 mg. The batchwise sample is subjected to ultrasonic vibrations for a maximum period of 8 h. Before the sample is charged into the sump of the motorbike, it is subjected to additional 20 h of mixing using the sonicator.

#### Kinematic viscosity tests

The viscosity of a lubricant is closely related to its ability to reduce friction. If the lubricant is too thick, then it will require a lot of energy to move the surfaces, and if it is too thin, then the surfaces will rub and the friction will increase. Viscosity index indicates the variation of viscosity with temperature. The best oils (with the highest VI) will not vary much in viscosity over such a temperature range and therefore will perform well throughout. A high value (normally >90) of viscosity index is an indicator of good lubricating oil.

The samples were tested for kinematic viscosity and viscosity index of the oil is calculated. Table 1. gives the values of viscosity at 40 and 100°C and viscosity index.

**Table 1:** Results of test for kinematic viscosity of different samples

Sample	Viscosity @40°C cst	Viscosity @100°C cst	Viscosity index
Racer 4	138.8	15.68	118
Racer-4 + 0.05% Cu	135.7	15.33	116
Racer-4 + 0.1% Cu	141	15.84	117
Racer-4 + 0.2% Cu	144.77	15.9	116
Racer-4 + 0.05% TiO_2_	136.23	14.47	106

From the results it can be noted that there is a slight increase in the kinematic viscosity with the addition of copper nanoparticles. It can also be observed that there is a reduction in the kinematic viscosity with the addition of TiO_2 _nanoparticles. Although there is a slight change in kinematic viscosities of oil samples with the addition of copper and TiO_2 _nanoparticles, there is no change in the viscosity index which remains in the high viscosity index region (>90). It can be concluded that the influence of viscosity on mileage of the motor bike is minimal and negligible at lower concentrations of nanoparticles.

### Tests results

Detailed tests are programmed on a stationary motorbike for the ranges of parameters listed as entries in Table 2.

**Table 2:** Details of ranges of test data

S. No	Lubricant	Speed (kmph)	Load (N)
1	Racer-4	40-60	10-50
2	Racer-4 + 0.05% Cu	40-60	20-80
3	Racer-4 + 0.1% Cu	40-60	20-80
4	Racer-4 + 0.2% Cu	40-60	20-80
5	Racer-4 + 0.05% TiO_2_	40-60	20-80

### Analysis of the test data

Analysis of the test results is quite complex since the rotating and reciprocating components in a mobile I.C engine are many, and these cannot be comprehensively described in the framework of a physical model. Hence, it can be described as a thermal system, following the principles of thermodynamics. The input thermal energy due to combustion of the fuel is partially utilized to do mechanical work to create mobility at a certain velocity under specified load conditions on the wheel. The heat balance sheet cannot be accurately drawn because of lack of information regarding frictional losses, thermal losses from the exhaust of the burnt gases, and other unaccounted losses. They cannot be separately segregated for a stationary vehicle. The best alternative is to conduct as many tests as possible and subject the data for statistical regression analysis.

The data are subjected to regression analysis as follows:

1. For the case with Racer-4

The fuel consumption *f*_c _is considered as(1)

where *F *is considered as a second-degree polynomial in the variable [*V^a ^P^b^*] and

where *a*, *b*, *A*_0_, *A*_1_, and *A*_2 _are constants to be determined by applying regression to the test data;

*f*_c _is the fuel consumption in (kg/h);

*V *is the linear velocity of the wheel (m/s); and

*P *is the brake power in (kW).

2. For the case with Racer-4 + Cu nanoparticles

where *F *is again considered as a second-degree polynomial in the variable 

where(2)

where *φ *is the percentage mass concentration of the nano component added into Racer-4.

The brake thermal efficiency can be computed from the relationship(3)

where *λ *is the calorific value of the (fuel kJ/kg).

The comprehensive data shown in Table 1 is subjected to nonlinear regression, and the results are shown in Figures 3 to 14

**Figure 3:**
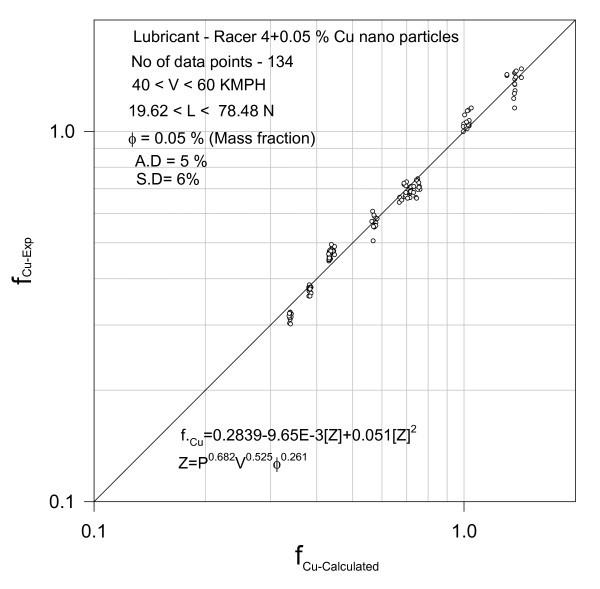
**Validation of the correlation**.

**Figure 4:**
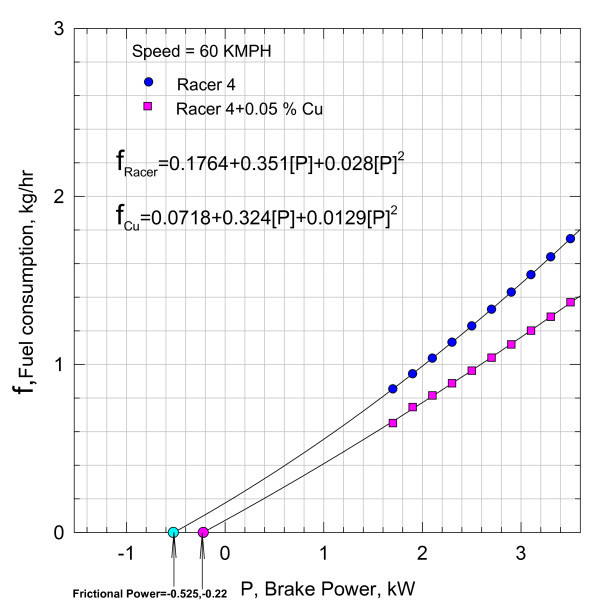
**Variation of fuel consumption with break power**.

**Figure 5:**
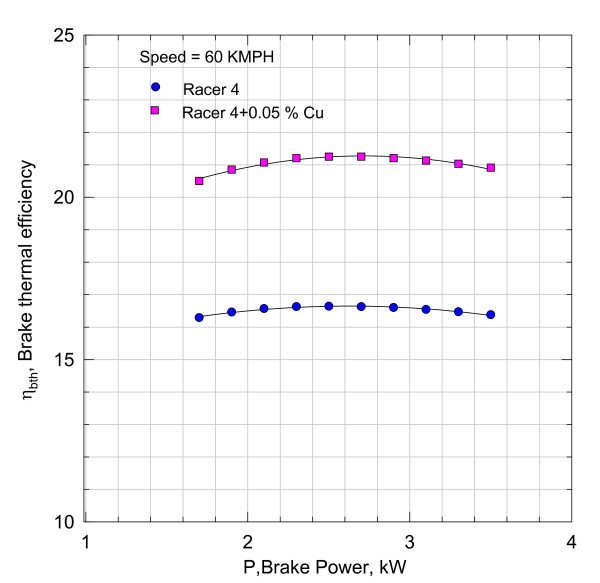
**Variation of brake thermal efficiency with brake power**.

**Figure 6:**
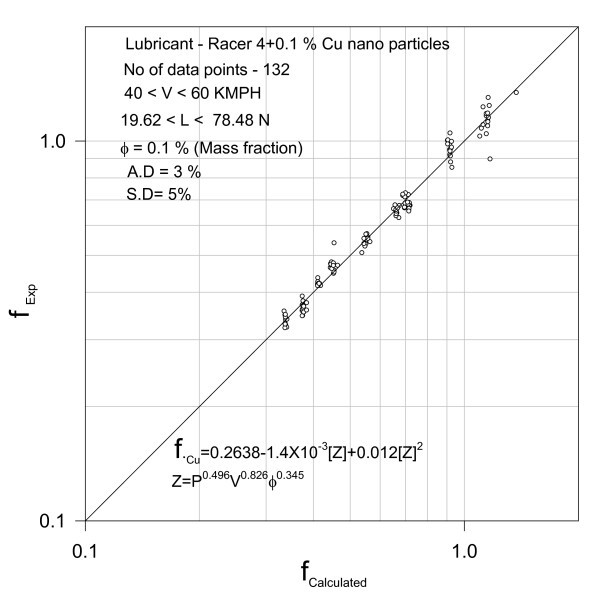
**Validation of correlation**.

**Figure 7:**
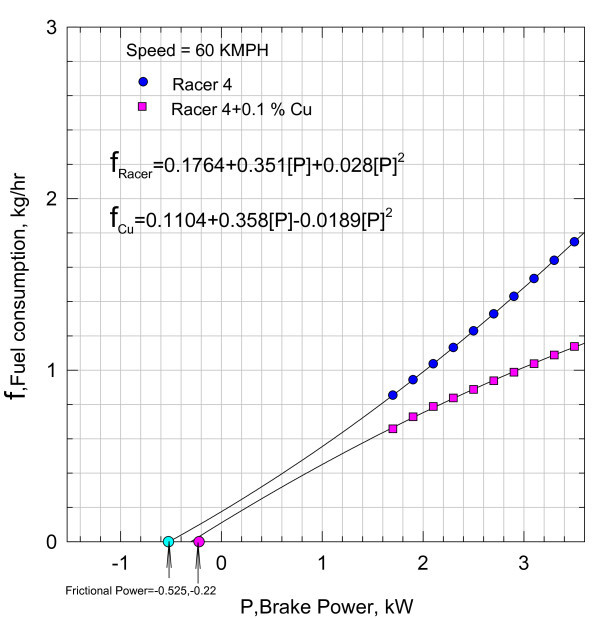
**Variation of fuel consumption with break power**.

**Figure 8:**
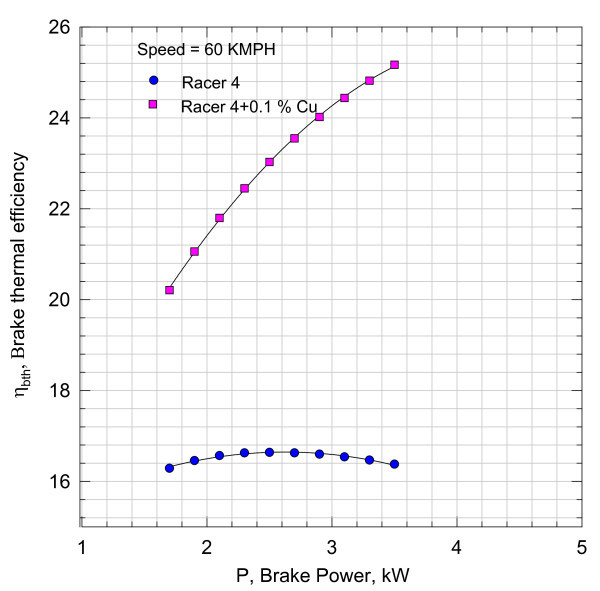
**Variation of break thermal efficiency with break power**.

**Figure 9:**
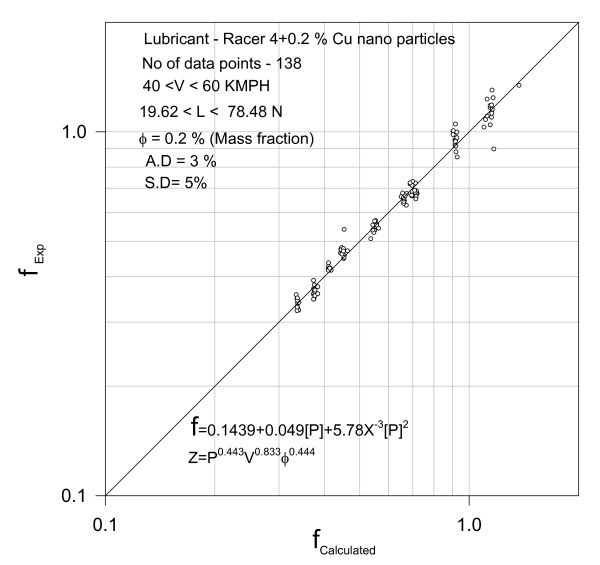
**Performance of the engine with Racer-4 + Cu nano particles as lubricant**.

**Figure 10:**
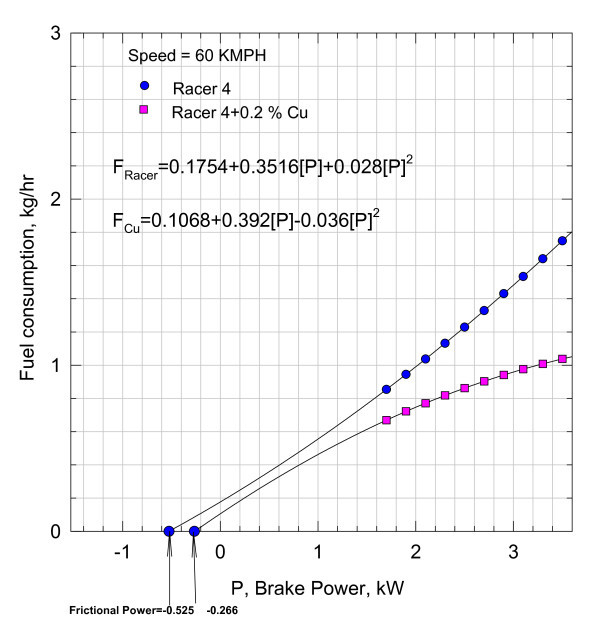
**Variation of fuel consumption with break power**.

**Figure 11:**
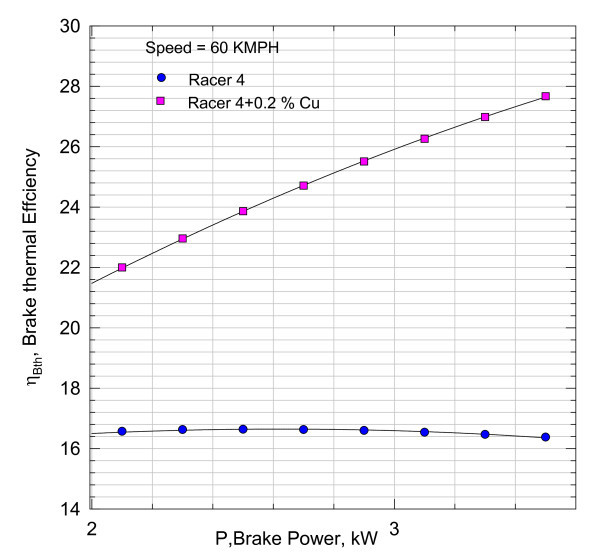
**Variation of break thermal efficiency with break power**.

**Figure 12:**
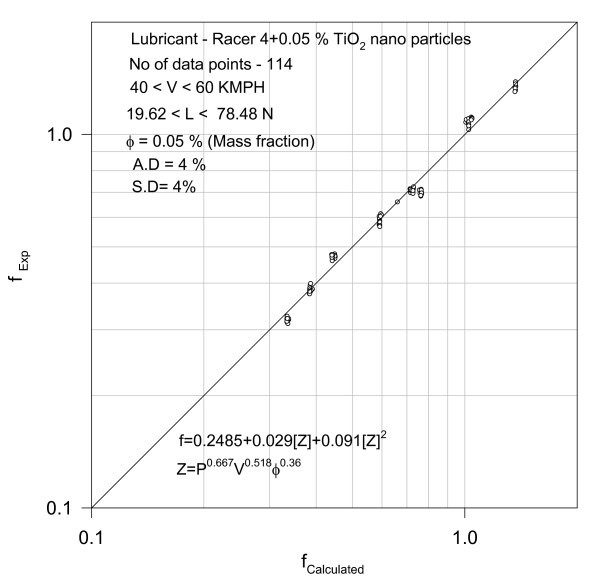
**Validation of correlation**.

**Figure 13:**
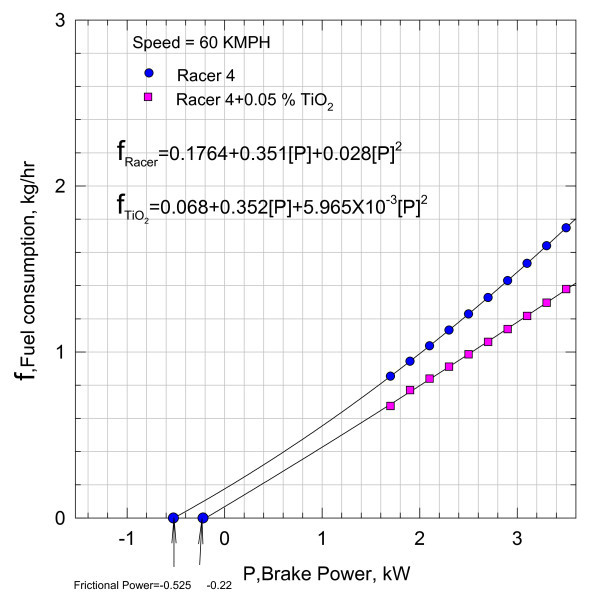
**Variation of fuel consumption with break power**.

**Figure 14:**
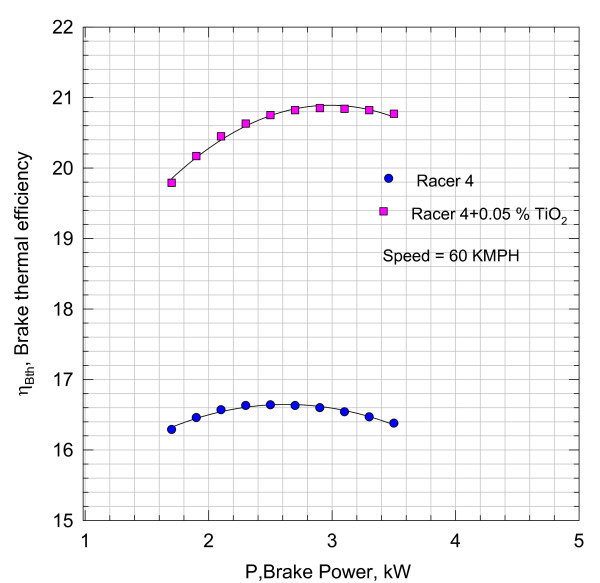
**Variation of break thermal efficiency with break power**.

### The results of the analyses for various cases

(1) Lubricant Racer-4 + 0.05% Cu

Results of regression yielded a polynomial as follows.(4)

The test data 114 points appearing as obvious from Figure 3 could be correlated by second-degree polynomial Equation (4) with an average deviation of 4% and a standard deviation of 4%.

Equation (4) indicating the fuel consumption as a function of brake power is plotted in Figure 4 at a speed of 60 kmph with 0.05% of Nano Cu in the lubricant.

The fuel consumption can be represented by a second-degree polynomial as follows:(5)

The fuel consumption of the motorbike with pure lubricant at a speed of 60 kmph is also shown plotted in Figure 4, and the functional variation is given by the relationship:(6)

Functional relationships Equations (5) and (6), i.e., *f*_Cu_, and f_Racer _are, respectively, further extended to cut the abscissa at -0.525 and -0.22 kW. Analytically, Equations (5) and (6) are subjected to Newton-Raphson method of analysis to check the correctness of the intercepts on the abscissa. The agreement with the values shown in the plot is very satisfactory, justifying the continuity of the functions, i.e., Equations (5) and (6).

It can be inferred that the addition of nano Cu reduced the frictional component substantially. However, these magnitudes include the heat losses from the cylinder and other unaccounted for losses. With the aid of Equation (3), the variation of brake thermal efficiency *η*_brake _with brake power is plotted in Figure 5.

The results shown in Figure 5 indicate that there is 4-5% rise in brake thermal efficiency with the addition of Cu Nano into the Racer-4 lubricant. Thus, the increase in brake thermal efficiency will lead to fuel conservation.

Similar mathematical analysis is carried out for the whole range of compositions, and the results are furnished further.

2. Racer-4 + 0.1% of Cu.

Figure 6 depicts that the test data could be satisfactorily correlated. The variation of fuel consumption with the variable *Z *is represented by the relationship:(7)

where *Z *= *P*_B_^0.496^*V*^0.0.826^*φ*^0.345^.

132 test results are correlated with an average deviation of 3% and a standard deviation of 5%. Further, variation of fuel consumption with brake power at 60 kmph is given by the second-degree polynomial in brake power, *P *(see Figure 7)(8)

By comparing these brake thermal efficiencies values of Figure 5 for 0.05% Cu Nano sample with those in Figure 8, it can be seen that increase in brake thermal efficiency at higher loads is found to be more marked. The efficiency characteristics shown in Figure 8 have a steeper gradient indicating better performance with load with 0.1% Cu Nano sample.

3. Racer-4 + 0.2% of Cu

The following relationships are obtained for this combination:(9)

where *Z *= *P*^0.443^*V*^0.83326^*φ*^0.444^

The correlation for 138 data points as shown in Figure 9 is achieved with an average deviation of 3% and a standard deviation of 5%.

At 60 kmph, a typical fuel consumption variation with power *P *is given in Figure 10.(10)

The engine is found to perform better at higher loads for this sample as can be noticed from Figure 10&11.

4. Racer-4 + 0.05% TiO_2_

The performance of the bike with a different type of nano TiO_2 _with 0.05% mass concentration in the racer is further investigated. The total number of tests is 114 and when subjected to regression (see Figure 12), it resulted in following relationship with an average deviation of 4% and a standard deviation of 4%(11)

where *Z *= *P*^0.667^*V*^0.518^*φ*^0.36^

At 60 kmph, the fuel consumption is given by the relationship (see Figure 13)(12)

However, the variation in brake thermal efficiency with brake power for lubricant with TiO_2 _sample is not as profoundly affected as can be seen from Figures 8 and 14 of nano lubricants with Cu and TiO_2_.

**Figure 15:**
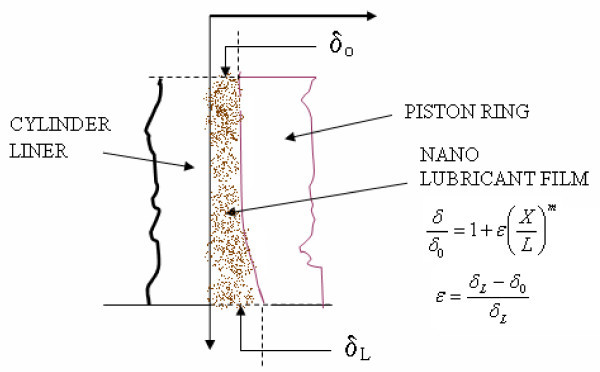
**Configuration of lubricant film**.

The results in Figures 7, 10, and 13 indicate that the frictional power is profoundly influenced due to the inclusion of nano Cu and TiO_2 _in the lubricant. The reason for such a decrease in the frictional power can be due to two factors, viz., either due to frictional coefficient or may be due to the geometric changes in the lubricant film gap thickness. Hence, to establish the plausible reasons, the problem is conceptually formulated with the aid of hydrodynamic lubrication theory. In practice, the rubbing surfaces between the liner and the piston ring cannot be parallel but the gap in between is varying with the lubricant medium in the film facilitating load bearing. Hence, subsequently from theoretical considerations, the likely reasons are investigated. In Figure 15, the lubricant film is geometrically idealized, and the equation of motion of the lubricant film is defined considering the viscous forces and pressure forces.

**Figure 16:**
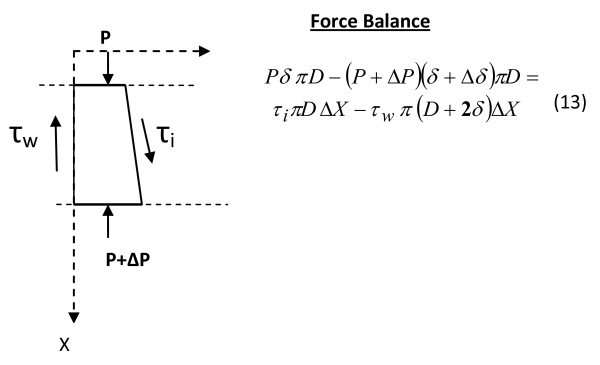
**Forces on the fluid element**.

Equation (13) (shown in Figure 16.) after simplification and proper arrangement with the assumption |*τ*_i_| = |*τ*_w_| yields the force balance in differential form as follows:(14)

The boundary condition for solving the differential equation (14) is that at(15)

*F*_r _is the friction factor parameter defined by the term 

where *P*_m _is the mean effective pressure acting on the piston head, *V *is the mean velocity of the piston, ε, is the gap factor between the liner and the piston rings, which is defined as 

 where *L *is the length of the stroke, and *m *is the variable exponent defining the gap profile between the liner and the piston ring.

The differential equation contains two parameters, *F*_r _and ε and by changing these terms, we need to evaluate the frictional power variation.

### Evaluation of frictional power

The frictional power *F*_P _can be estimated as follows:(16)

Thus, the values *F*_P _can be estimated from the pressure profile(17)

### Solution of Equation (14)

Equation (14) is written in finite difference form as follows:

*I *is the variable node with *I *varying *I *= 1 to [*J *+ 1] nodes for the range 0 <*X*^+ ^< 1

Thus,(18)

where 

Equation (16) can be computed from(19)

**Figure 17:**
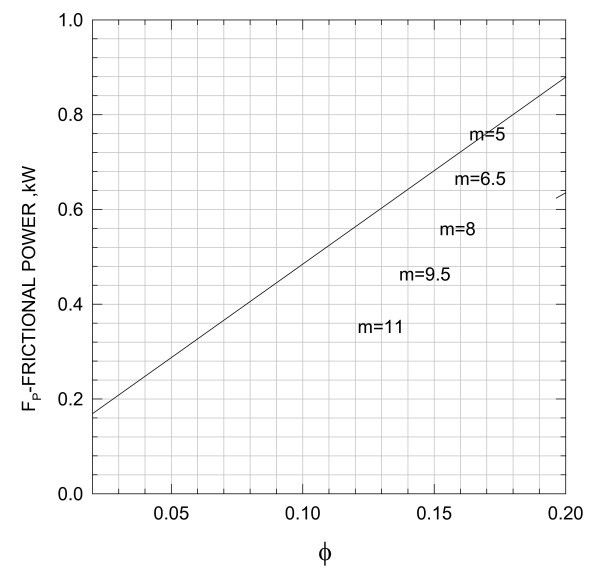
**Variation of Frictional power with geometry of liquid film**.

**Figure 18:**
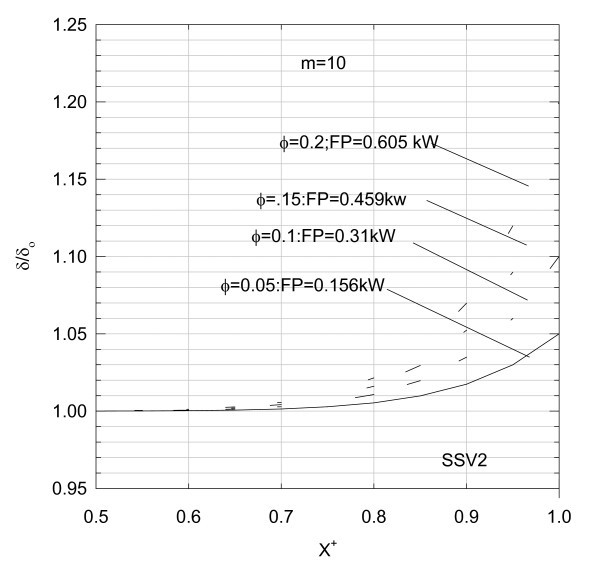
**Variation of liquid film thickness between the piston ring and cylinder liner**.

**Figure 19:**
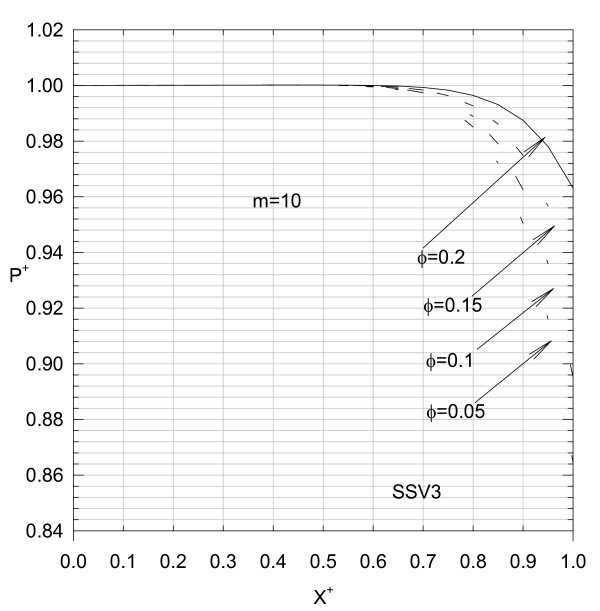
**Variation of Dimensionless pressure in the liquid film**.

Thus, for different values of *F*_r_, *m*, and ε computer runs are generated, and the results are shown in Figures 17 to 19.

**Figure 20:**
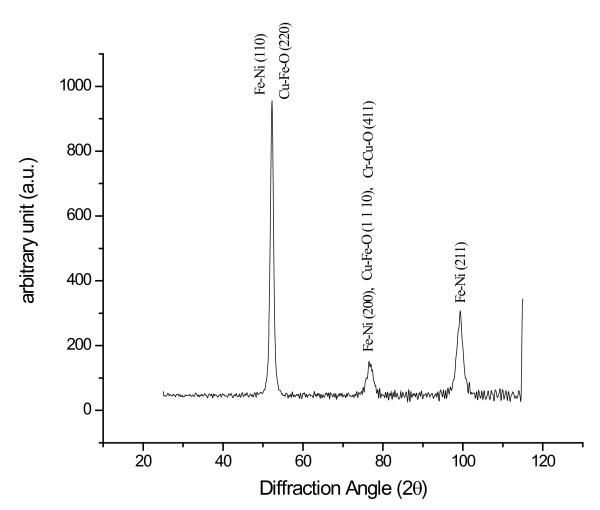
**XRD analysis of the ball specimen subjected to ASTM 4 ball wear test**.

After conducting ASTM 4 ball wear tests, the balls of wear test were subjected to XRD analysis for possible deposition of copper nanoparticle. X-ray-scattering techniques are a family of nondestructive analytic techniques which reveal information about the crystallographic structure, chemical composition, and physical properties of materials and thin films. These techniques are based on observing the scattered intensity of an X-ray beam hitting a sample as a function of incident and scattered angle, polarization, and wavelength or energy. The following graph shown in Figure 20 depicts the XRD analysis.

From the graph, it can be noted that along with iron(Fe), nickel(Ni), chromium (Cr), oxygen(O), and copper (Cu) can also be seen at peaks of the plot. This suggests that small amount of copper nanoparticles deposit on the surface of the ball and form a protective coating (Mending effect),thereby offering resistance to wear and reducing the friction.

## Results of exhaust gas analysis

Though the present tests and studies are limited to nano lubricants with Cu and TiO_2_, the typical exhaust gas analysis is presented. It is premature to comment on the exhaust gas analysis. However, the results in the last three columns indicating CO_2_, HC, and NO_*x *_seem to be marginally influenced. To conclude, further detailed data must be collected and analyzed. Besides, the nano is not directly introduced into the fuel to alter the combustion characteristics (Table 3).

**Table 3:** Results of exhaust gas analysis

Lubricant	CO (%)	CO_2 _(%)	HC (ppm)	NO_*x*_(ppm)
Racer-4 + 0.1% Cu	2.672	7.15	489	15
Racer-4 + 0.2% Cu	2.582	6.89	513	34
Racer-4 + 0.05% Cu	2.873	7.01	446	28
Racer-4	2.587	8.36	485	71
Racer-4 + 0.05% TiO_2_	2.595	3.28	553	0

## Conclusions

The nano lubricants dispersed with nano Cu in 0.05, 0.1, and 0.2% mass fractions yielded the enhanced brake thermal efficiencies in relation to the performance of an engine with Racer-4 of HPCL. The gradients in the characteristics for nano lubricants are found to be steeper. The 4-7% rise in thermal efficiency as seen in Figures 8, 11, and 14 in relation to the performance of the bike with pure lubricant is a promising feature in terms of the fuel economy. It can also be inferred that the bike can accept higher loads at speeds of about 60 kmph or more with nano lubricants other than Racer-4. 0.1% nano Cu-dispersed lubricant which is found to yield better results than with mass concentrations of 0.05 and 0.2%.

Introduction of nanonoparticles into the lubricant effectively reduces the overall frictional power. This aspect is clear from the intercepts of fuel consumption characteristics with the brake power abscissa. The frictional results could be checked analytically with the Newton-Raphson method. The reduction in frictional power may be due to substantial decrease in coefficient of friction between reciprocating and rotating parts. However, this aspect is to be further ascertained.

Though the results with the 0.05% TiO_2 _Nano indicate increase in brake thermal efficiency, the performance of the engine is not on par with the other lubricant as with nano Cu lubricant.

The assessment of life of the nano lubricant with Cu and TiO_2 _in terms of total mileage is still a matter of further examination. It is found to yield fuel economy promoting fuel conservation in view of the increasing number of two- and four-stroke motorbikes on the road. The technology is still to be developed to inhibit unwanted agglomeration of Cu nano over a period of time in the lubricant. The study indicates that if nanochemistry offers a solution to this problem, the dispersion with 20-50 nm Cu and TiO_2 _stands fairly a good chance in the lubrication technology as applied to I.C. engines in general.

## Abbreviations

**List of symbols ***A*_0_, *A*_1_, *A*_2_: Constants in the polynomial equations; : Interfacial area in the cylinder (m^2^); *a*: Constant; *b *: Constant; *D*: Cylinder diameter (m); *f*: Fuel consumption (kg/h); *F*_P_: Friction power (kW); *F*_r _: The friction factor parameter; *L*: Length of the stroke (m); *m*: The variable exponent defining the gap profile; *N*: No. of revolutions of the rollers; *P*: Pressure (N/m^2^); *P*^+^: Dimensionless pressure parameter; *P*_B_: Brake power (W or kW); *V*: Linear velocity of the wheel (m/s); *W*: Load applied on the wheel (kgf or N); *X*: Distance along the stroke (m); *X*^+^: Dimensionless distance along the stroke **Roman letters **δ: Lubrication film thickness at any point (m); δ_O_: Lubrication film thickness at one edge (m); δ_L_: Lubrication film thickness at other edge (m); ε: The gap factor between the liner and the piston rings; φ: Mass concentration ; η: Efficiency; τ: Shear force (N/m^2^); λ: Calorific value of fuel (kJ/kg) **Subscripts **Brake Brake thermal efficiency Cu: Copper; i: Interfacial; m: Mean effective; Racer: With Racer-4 as lubricant; TiO_2_: Titanium dioxide; w: Wall.

## Competing interests

The authors declare that they have no competing interests.

## Authors' contributions

VS is the Principal Investigator of the HPCL sponsored project and has done extensive experimentation on lubricants dispersed with nano particles. Experimentation has been done to determine the physico-chemical properties, friction & wear characteristics and performance characteristics of the motor bike to check the effectiveness of specified nano lubricant. PKS is the technical adviser of the project and has done modeling of the experimental results obtained in experimentation. He performed the statistical and regression analysis in the project. VDR is an external expert and adviser of the project. He has been assisting by the way of course correction during the progress of the project. AKK is a research scholar who worked in the project assisting the Principal Investigator
